# Application-Layer Time Synchronization and Data Alignment Method for Multichannel Biosignal Sensors Using BLE Protocol

**DOI:** 10.3390/s23083954

**Published:** 2023-04-13

**Authors:** Jianan Li, Eric Quintin, He Wang, Benjamin E. McDonald, Todd R. Farrell, Xinming Huang, Edward A. Clancy

**Affiliations:** 1 Worcester Polytechnic Institute, Worcester, MA 01609, USA; jli6@wpi.edu (J.L.); hwang9@wpi.edu (H.W.); xhuang@wpi.edu (X.H.); 2 Liberating Technologies, Inc., Holliston, MA 01746, USA; lti-rd@liberatingtech.com (E.Q.); benjamin.mcdonald@liberatingtech.com (B.E.M.); todd.farrell@liberatingtech.com (T.R.F.)

**Keywords:** Bluetooth low energy (BLE), biosensor, time synchronization, wireless sensor network, Internet of things (IoT)

## Abstract

Wearable wireless biomedical sensors have emerged as a rapidly growing research field. For many biomedical signals, multiple sensors distributed about the body without local wired connections are required. However, designing multisite systems at low cost with low latency and high precision time synchronization of acquired data is an unsolved problem. Current solutions use custom wireless protocols or extra hardware for synchronization, forming custom systems with high power consumption that prohibit migration between commercial microcontrollers. We aimed to develop a better solution. We successfully developed a low-latency, Bluetooth low energy (BLE)-based data alignment method, implemented in the BLE application layer, making it transferable between manufacturer devices. The time synchronization method was tested on two commercial BLE platforms by inputting common sinusoidal input signals (over a range of frequencies) to evaluate time alignment performance between two independent peripheral nodes. Our best time synchronization and data alignment method achieved absolute time differences of 69 ± 71 μs for a Texas Instruments (TI) platform and 477 ± 490 μs for a Nordic platform. Their 95th percentile absolute errors were more comparable—under 1.8 ms for each. Our method is transferable between commercial microcontrollers and is sufficient for many biomedical applications.

## 1. Introduction

Recent development of wireless technologies and low-power wireless transmission protocols have paved the way for using wireless biosensors to continuously monitor human biosignals [1,2,3,4,5,6,7]. The use of low-power BLE (Bluetooth low energy), ZigBee, and Wi-Fi (IEEE 802.11ah or Wi-Fi HaLow) is increasing rapidly in Internet of things applications, wearable wireless systems for health monitoring, and related areas [8,9,10,11,12,13]. EEG (electroencephalogram), ECG (electrocardiogram), and EMG (electromyogram) are frequently used in medical and health applications. These applications require low power consumption, low latency, and high bandwidth data transmission. Comparing different low-power wireless transmission protocols, BLE has much lower power consumption (~1 mA compared to ~10 mA for low-power Wi-Fi), as well as a high transmit rate of 2 Mbps physical layer compared to ZigBee’s 250 kbps [14,15,16,17,18]. The faster transmit rate supports higher bandwidths and lowers latency. Although custom wireless protocols might exceed this performance, they are difficult and expensive to develop and upgrade, whereas improved commercial off-the-shelf device performance occurs at a rapid pace. Hence, we sought to develop wearable systems based on the standard BLE 5.0 wireless transmission protocol.

Most biosignal applications require multiple signal channels [19]. For example, two EMG channels are typically required for myoelectric control of a prosthesis, our own primary area of interest. The minimum sampling rate of such applications is high, e.g., 1000–2000 Hz for EMG [20], as high as 500 Hz for EEG [21], and 250–360 Hz for ECG [22,23]. Currently, most wireless sensor systems either use one combined peripheral data sender hard-wired to multiple sensors [24] or use custom wireless protocols to achieve high throughput and low latency (Trigno^®^ Research+ System, Delsys Inc, Boston, MA, USA). Combining sensors at a peripheral node does not eliminate wires locally attached across the body (thus, not fully wireless) and can make it hard to locate all electrodes at their desired positions [25,26,27]. Fully distributed wireless multichannel peripheral nodes would make it easier to select the best sensor sites. Furthermore, custom protocols make it hard to migrate from one platform to another to take advantage of ongoing electronic device improvements. In addition, custom protocols are harder to share within the research community.

Traditionally, wired multichannel data acquisition systems using a multichannel analog-to-digital converter (ADC) inherently time-synchronize all channels to within one sample period. However, with distinct wireless nodes, each ADC is controlled via its local peripheral clock which operates at a slightly different sampling rate and sampling phase [28]. In addition, each clock rate may drift over time [29]. Thus, a method is needed to synchronize time between wireless devices, and then use that time synchronization to align multiplexed data at the central node [30,31,32]. For the Bluetooth, ZigBee, and Wi-Fi protocols, native time synchronization methods are not accurate enough for high-sample-rate biosignals [33].

In this paper, we describe a BLE 5.0 time synchronization and data transmitting system that is programmed at the application layer, demonstrated via two peripheral devices and one central device, and tailored for low-latency high-throughput applications. The method is expandable to more peripheral devices. Two peripheral devices concurrently transmitted ADC samples at 1000 Hz sampling rate, with latency ≤30 ms. Time synchronization was applied on the basis of paired timestamps from the peripheral and central nodes, with random timing variation reduced via a linear least squares regression algorithm. The central node (or an offline algorithm, in our study) then multiplexes the received data from the peripheral nodes, with proper time alignment. Our method was implemented separately on two common microcontroller platforms (TI and Nordic), demonstrating its transferability. No modification of the underlying transmission protocols and no extra hardware were needed.

The primary goal of this study was to validate a novel BLE time synchronization and alignment method that is low-latency, high-throughput, and transferable between manufacturer microcontroller platforms (i.e., implemented at the application layer). We characterized its performance vs. the number of timestamp pairs and the timestamp update interval used by the regression algorithm. We evaluated these parameters on two microcontroller platforms. We hypothesized that too few timestamp pairs used in the regression would lead to higher data alignment errors, and that too many timestamp pairs would not follow real changes in central vs. peripheral timing. Similarly, too short of a timestamp update interval was hypothesized to provide averaging over too short of a time duration (thus producing poorer data alignment) while too long of an interval would not follow actual timing changes.

The sections which follow begin (Section 2) by reviewing background information on existing time synchronization methods and relevant characteristics of BLE and the BLE-based microcontrollers used in this research. Section 3 describes our approach to time synchronization and data alignment. Section 4 presents our laboratory experiments that tested the performance of these algorithms as a function of selectable system parameters. Section 5 gives the results of these tests. The paper ends with a discussion and some conclusions.

## 2. Background

### 2.1. Existing Time Synchronization Methods and Their Limitations for This Application

As summarized in Table 1, several methods have been introduced to achieve better time synchronization when using BLE (see reviews in [34,35,36]). One method is based on the Bluetooth beacon role [37,38,39], in which a central node broadcasts clock information that is received near-simultaneously by all listening peripheral nodes (and with low latency). All peripheral nodes then synchronize to the central node. Beacon transmissions are repeated to maintain synchronization over time. However, when the central node broadcasts, it cannot receive data from different peripheral nodes in real time. Thus, this beacon role method is not suitable for high-throughput, low-latency applications. Another method uses extra hardware to detect the onset of antenna activation when transmissions are initiated [40], achieving a synchronization precision of 9 ± 17 μs. However, additional hardware must be included, some extra battery power is always consumed, and such custom hardware is not readily upgradable. For daily health monitoring, less power consumption (and, thus, longer battery life) is desired. For typical wireless nodes with a 20 mAh battery, the additional hardware can preclude a desired battery life of 16 h (for example). Another recent method used BLE non-connectable non-scannable undirected advertising (BLE “ADV_NONCONN_IND”) to reset peripheral clocks to achieve time synchronization [41]. However, this technique also did not demonstrate a manner in which to maintain synchronization of streaming ADC data from peripheral nodes [42]. Hence, there is a need for a BLE time synchronization and data alignment method that is low-latency, high-throughput, and implemented at the application layer (thus, transferable across devices).

There exist other more traditional methods for wired and wireless networks. However, these methods are not considered appropriate for a high-bandwidth, low-latency, and continuous data stream using BLE. The NTP (network time protocol) [43], for instance, was designed for large-node wired systems and employs a complex hierarchy of nodes and methods for rejecting anomalous synchronization information. NTP is not considered suitable for use in BLE systems, given its substantial volume of synchronization messages, high computation, and low energy inefficiency. TTS (traditional time synchronization) [44] assumes that two-way messaging between wireless nodes can be executed within a short timeframe, rendering this technique unsuitable for BLE systems. Message exchange times in BLE are not firmly controlled. The time-synch protocol for sensor networks (TPSN) [45,46] and flooded time synch protocol (FTSP) [31] are programmed at the MAC (medium access control) layer and not the application layer; thus, they are also unsuitable for this application.

### 2.2. Relevant Bluetooth Low Energy Characteristics

IEEE 802.15.1 (Bluetooth) is a wireless technology standard used for short-distance applications, which operates in the 2.402 GHz to 2.480 GHz band. BLE was introduced to overcome some of the limitations of the standard Bluetooth version, such as high power consumption, low packet size, and high link reestablish time. BLE 5.0 [47], used in this research, has a transmit rate of 2 Mbps, maximum data packet size of 251 bytes (plus four header bytes), average power consumption of 0.01–0.5 W, and connection latency of 6 ms in a non-connection state (and even lower in a connection state).

The BLE protocol stack consists of two main levels: the controller and the host. The controller is the lower level which mediates BLE packet sending and receiving, differing from platform to platform. To ensure transferability at the application level, the controller level was not modified. The host level, which is the application layer, can more easily migrate from one platform to another. We implemented synchronization and data alignment in “C” code at this level. The host–controller interface (HCI) is used to communicate between the two levels, exchanging packetized data. Within the host level, the generic access profile (GAP) must run in one of four roles: broadcaster, observer, peripheral, or central. As our application needs to transmit data bidirectionally (central timestamp data from central to peripheral; ADC data and peripheral timestamp from peripheral to central), the central role and peripheral role are required.

In the BLE peripheral–central role, the central and peripheral nodes cannot remain continuously connected when multiple peripheral nodes are used. A connection interval ranges from 7.5 ms to 4 s, with a gap of 1.25 ms required between assigned time slots. If multiple peripheral nodes pair with one central node, each peripheral node’s connection interval is set to their connection setting’s least common multiple. A connection interval of 15 ms was used for the two-peripheral implementation on the TI platform, and a connection interval of 10 ms was used for the Nordic platform. These were the smallest intervals supported by the respective platforms.

### 2.3. The TI and Nordic Wireless Microcontrollers

The TI CC2640R2f is a BLE module manufactured by Texas Instruments (TI). It features Bluetooth 5.0 and a 32 bit 48 MHz ARM Cortex^®^-M3 processor. The integrated antenna has a maximum transmit (TX) power of +5 dBm and a receiver sensitivity of −97 dBm. It requires a 5.9 mA receive (RX) current, a 6.1 mA TX current at 0 dBm, and a 9.1 mA TX current at +5 dBm, all from a 1.8–3.8 V supply. It has a built-in eight-channel 12 bit 200 k samples/s ADC. However, its 28 kB static random-access memory (SRAM) was only sufficient in our work when this microcontroller was used as a peripheral node. Hence, our TI-based implementations used a TI CC2642 as the central node. This model has an 80 kB SRAM and slightly higher RX (6.9 mA) and TX current consumption (7.3 mA at 0 dBm and 9.6 mA at +5 dBm), from a 1.8–3.8 V supply. Overall, due to these characteristics and its small size (7 mm × 7 mm), it is an excellent selection for wireless biosensor systems.

To evaluate and demonstrate the ease of migration of our time synchronization methods across platforms, a competitive BLE module was selected. The Nordic Semiconductor nRF52840 also features Bluetooth 5.0 with a 32 bit 64 MHz ARM Cortex^®^-M4 processor. Its current consumption is 4.6 mA RX and 4.8 mA TX, from a 1.7–5.5 V supply. It also has a built-in 12 bit 200 k samples/s ADC and is equipped with a large RAM size of 256 kB. With even lower power consumption and similar integrated features, it is also an excellent test platform choice.

## 3. Time Synchronization and Data Alignment Methods

### 3.1. System Architecture

In this research, we assembled two BLE wireless biosignal sensor bench-top systems to prototype our time synchronization and data transfer method. In each case, only ADC data and timestamps were collected in real time on the microcontrollers (i.e., data logging). Time synchronization and data alignment were then completed offline. In this manner, the same data could be used to evaluate multiple parameter combinations of the time synchronization method, which facilitates a more robust comparison. In embedded applications, the complete time synchronization and data alignment algorithm would be implemented online in the peripheral and/or the central nodes of the system.

**Figure 1:**
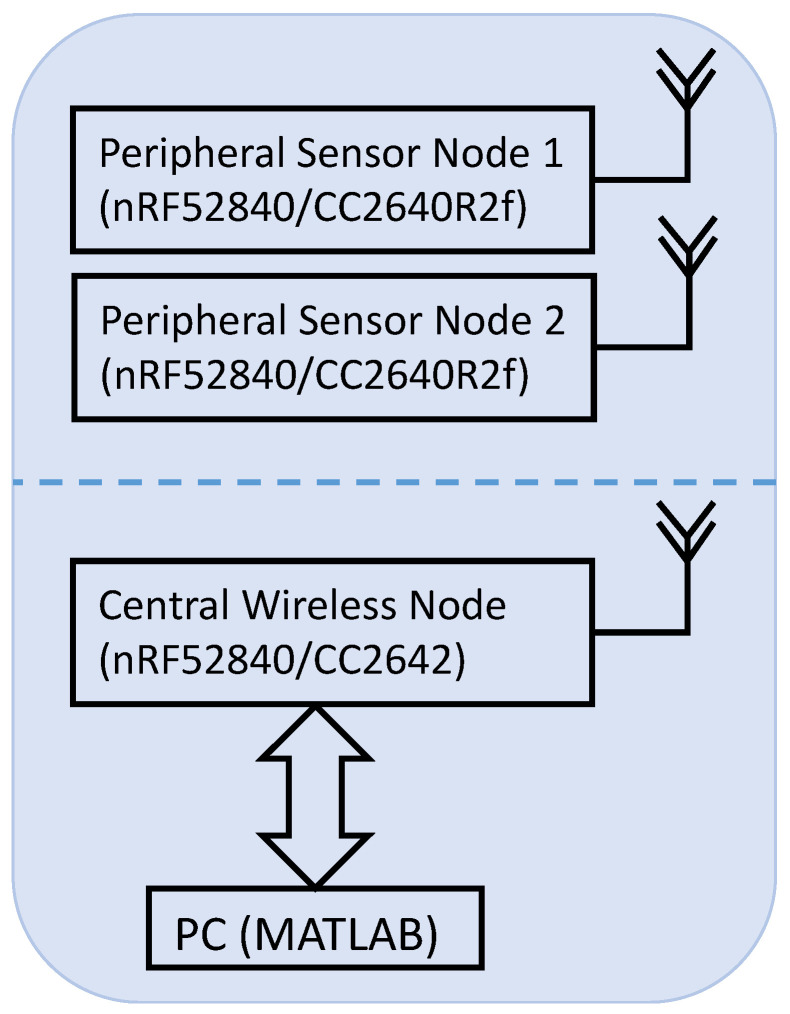
System diagram for both TI and Nordic platforms.

The first system (Figure 1) was based on a TI BLE development board platform. Variants of these development boards, utilizing the same active hardware, are available in smaller packages for embedded system use. Use of the full-size development board facilitated rapid prototyping. The first part of the system comprised two peripheral biosensor nodes (one sensor per node), each using TI CC2640R2f boards with built-in 12-bit ADC to sample the biosignals. These peripheral nodes wirelessly transmitted data and timestamps to one central node utilizing a TI CC2642 development board. For this research study, the central node logged all data and timestamps to a PC using its UART port. Thereafter, data processing used MATLAB on a PC.

With only software modifications at the application layer, we implemented the second system (Figure 1) using Nordic BLE development boards. The architecture was similar to that of the TI-based system, except that both peripheral nodes and the central node used the same Nordic development board (nRF52840). All data were still streamed to the PC for offline analysis.

The bandwidth of most bioelectric signals is less than 500 Hz, with EMG having the largest range of 10–500 Hz [20,48]. According to the Nyquist–Shannon theorem, a sampling rate of 1000 Hz is required to correctly reconstruct a signal containing frequencies up to 500 Hz. Hence, we selected a 1000 Hz sampling rate. To achieve continuous transmission of ADC values without delay, a dual buffer structure was used for data collection on a peripheral node. When one buffer was full, an interrupt triggered its transmission, while the second buffer stored the new data without delay. The ADC automatically toggled incoming data storage back and forth between these buffers to achieve no missed ADC samples.

The connection interval (and ADC buffer duration) was 15 ms on the TI platform and 10 ms on the Nordic platform. During preliminary testing, these intervals were the minimum achieved without blocked transmissions in each respective platform.

### 3.2. Time Sychronization Method Design

#### 3.2.1. Generation of Timestamp Pairs and ADC Timestamps

All newly available ADC samples were transmitted in data packets at each connection interval (15 ms for TI; 10 ms for Nordic). Ideally, each connection interval would generate one ADC packet. However, timing variations between a peripheral and central clock can occasionally lead to either zero or two packets formed within a connection interval. BLE transmission from a peripheral clock is not synchronized directly with ADC packet readiness. Rather, the peripheral clock sets the timing of ADC conversion on a peripheral node, whereas the central node schedules BLE transmission. Regardless, as each new ADC packet is generated, it is placed in the peripheral node’s BLE transmit buffer for transmission to the central node during the next connection event. Once queued for transmission, the peripheral’s application layer software has no further access to the packet. Thus, packet receive time on the central node is a poor indication of ADC conversion time on a peripheral node. Transmission delay times unpredictably range from near zero up to one connection interval, or longer if blocked transmissions are automatically rescheduled for transmission during ensuing connection events. Hence, time synchronization based on arrival time on the central node has an uncertainty of up to several connection intervals (in our case, 10 or 15 ms per connection interval), which is too long for many applications. An alternative time synchronization method is necessary. One alternative is to use beacon transmissions (from the central node to all peripheral nodes) at system startup, thereby synchronizing clocks once (“single shot”) [37]. Data transmissions would begin thereafter, since they cannot run coincident with beacon transmissions. However, differences in clock rate, which are always present, would cause timing errors to accumulate over time. In addition, clock drift is not necessarily consistent throughout device operation, i.e., due to changes in temperature, vibrations, pressure, and other conditions [29]. As noted previously, repeated/continuous use of beacon transmissions is not consistent with continuous ADC sampling; hence, it is also not a time synchronization option.

In our approach, all data are moved between nodes using BLE notifications from within the application layer. A notification transmission, as opposed to a BLE indication, does not receive confirmation, thus minimizing delay and wireless transmission duration (at the risk of increased data loss). The basis of our time synchronization method is to generate time-synchronized (paired) central and peripheral timestamps. The more closely they are paired in time, the better. We found that the most reliable time fiducial occurred when initiated on the central node upon receiving a peripheral data packet. In particular, if the central node queried its timestamp clock immediately after peripheral data arrival (software-requested timestamps are available from within the application layer), then added one connection interval to this value, an excellent estimate of the arrival time was produced on that peripheral node of the ensuing central data packet BLE notification transmission (which was only transmitted on connection intervals in which paired timestamps were desired). The central data packet included this central timestamp, denoted $TS_{C}\left[m\right]$, where m indexes the timestamp pairs. Once this central data packet was received on the peripheral, it immediately queried its own timestamp clock (with this timestamp being denoted $TS_{P}\left[m\right]$, which is also available from within the application layer), forming a timestamp pair. The timestamp pair can be used on the peripheral node for data synchronization, or transmitted back to the central node in the next peripheral data packet for synchronization on the central node or on the PC (as applied herein). New timestamp pairs were not generated every connection interval (see below).

In addition, the ADC clock on the peripheral node ran asynchronously from the BLE sub-system. However, when the ADC completed converting data for a packet, a software interrupt was automatically generated at the application layer. Hence, the peripheral node immediately queried the timestamp clock again and associated this time with the final ADC sample for that packet. This timestamp was denoted $TS_{ADC}$.

#### 3.2.2. Timestamp Rollover Avoidance

The TI platform generates unsigned 32 bit integer timestamps, which count the number of 10 μs intervals since power-up. Thus, this timestamp rolls over every 11.93 h. The Nordic platform generates an unsigned 24 bit integer count of the number of 30.1 μs intervals since power-up, rolling over every 8.42 min. These rollovers are too short and would threaten robust real-time synchronization at rollover. We, therefore, re-stored each timestamp in an unsigned 64 bit integer, accounting for rollover when doing so (i.e., incrementing the 64 bit count through each rollover). The 64 bit timestamps were used thereafter and rolled over at durations greater than 5 million years. Use of 64 bit unsigned integers was convenient since many compilers for these embedded systems support them.

#### 3.2.3. Time Synchronization Model

The N most recent timestamp pairs ($TS_{C}\left[m\right]$ and $TS_{P}\left[m\right]$) were used in a linear least-squares clock synchronization method to continuously estimate central clock time. To understand this method, let $TS_{P}\left[m\right],\;0\leq m<N$ be the most recent peripheral node clock timestamps and $TS_{C}\left[m\right],\;0\leq m<N$ be the paired set of central node clock timestamps. The affine model that estimates central time on the basis of peripheral time is

(1) $${\hat{TS}}_{C}\left[m\right]=\beta_{0}+\beta_{1}\cdot TS_{P}\left[m\right]+\epsilon ,$$

where $\beta_{0}$ is the offset parameter, $\beta_{1}$ is the slope parameter, m is the timestamp index, and ϵ is a random error term. Since it is assumed that both clocks have reasonable time precision with slightly different drifting rate, it will be the case that the slope parameter $\beta_{1}$ has a value near 1.0 counts/count. The offset term $\beta_{0}$ can vary over the full range of the timestamp values and can be a negative value. With N timestamp pairs, we can estimate $\beta_{0}$ and $\beta_{1}$ via linear least squares [49] as follows:

(2) $$\left\{\begin{array}{ccc} \beta_{1} & = & \frac{N\cdot \Sigma_{PC}-\Sigma_{P}\cdot \Sigma_{C}}{N\cdot \Sigma_{PP}-\Sigma_{P}\cdot \Sigma_{P}}\\ \beta_{0} & = & \frac{\Sigma_{C}-\beta_{1}\cdot \Sigma_{P}}{N} \end{array},\right.\;$$

where $\Sigma_{PC}=\overset{N-1}{\underset{m=0}{\sum }}TS_{P}\left[m\right]\cdot TS_{C}\left[m\right]$, $\Sigma_{P}=\overset{N-1}{\underset{m=0}{\sum }}TS_{P}\left[m\right]$, etc. This least-squares approach has a low cost computationally and is a direct (noniterative) solution without issues of convergence.

**Figure 2:**
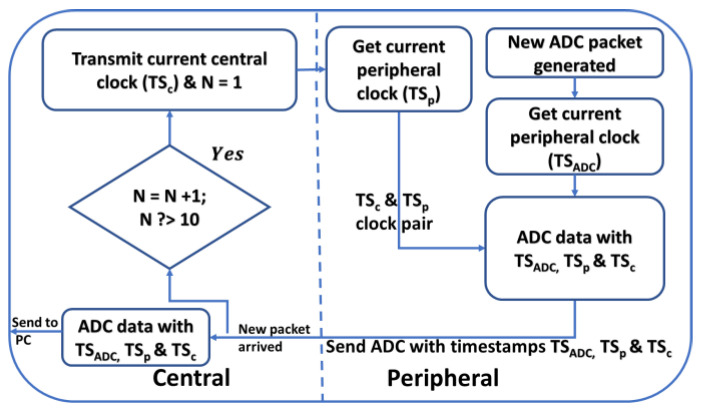
Flowchart of time synchronization method, using N = 10 as an example. Variables N, $TS_{C}$, $TS_{P}$, and $TS_{ADC}$ are defined in the text.

This model was updated as each new timestamp pair was received (or at a lower rate, if desired). For each data packet from the peripheral node, the most recent affine model was applied to the ADC timestamp ($TS_{ADC}$), producing an estimate of the central time corresponding to the last ADC sample. The central time corresponding to earlier samples in the packet was estimated using the sampling period. A flowchart of the time synchronization method is shown in Figure 2.

#### 3.2.4. ADC Data Latency

Note that the connection interval is the primary factor in setting minimum latency of each system. Shorter connection intervals lead to shorter latency. Latency can be segmented into the sum of three delays: (1) the time from ADC sampling to scheduling for transmission from the peripheral to the central node, (2) the subsequent time to transmit from the peripheral node and be received on the central node, and (3) the time on the central node to time-synchronize, data-align, and group samples with other peripheral streams.

The first of these delays is equal to the connection interval plus a small amount of processing time. It is equal to the connection interval since the data are not even scheduled for transmission until the final sample in the packet is acquired. This sample is already delayed by approximately one connection interval. This delay is relatively consistent from packet to packet.

The second delay varies depending on channel availability and assignment, and it can be longer if a transmission is blocked (and, thus, saved for transmission after the ensuing connection interval). The experimental system had few, if any, blocked transmissions. This delay can be quite variable, but tends to be a few ms in duration.

The third delay depends on the relative timing of the arrival of packets from other peripheral nodes. That is, the central node must wait for data to arrive from all peripheral nodes, but those data arrive serially. In the Nordic platform with two peripheral nodes, the transmissions can be arranged to alternate time slots between peripheral nodes, separated by one-half of the connection interval. Of course, there is some amount of time required for time synchronization and data alignment processing (again, relatively short in duration). This delay is at least the duration of one-half of a connection interval.

Taken together, a rough estimate of latency is approximately twice the connection interval, especially for the short-duration connection intervals used (and our avoidance of blocked transmissions). We did not make real-time latency measurements in this study.

### 3.3. Data Alignment Algorithm Design

**Figure 3:**
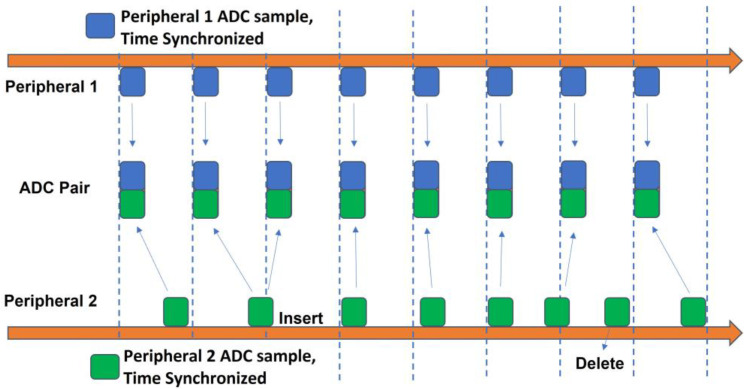
Illustration of ADC stream deletion and insertion of a sample, as needed, to maintain data alignment. The sample time corresponding to each peripheral 1 ADC sample is adjusted to the estimated central node time using its respective linear regression time synchronization model (which is based on paired timestamps—see Figure 2). ADC samples from peripheral 2 are similarly time-synchronized, using its respective model. Whenever too few ADC samples arrive from peripheral 2, an extra peripheral 2 sample is inserted (shown above). Whenever too many ADC samples arrive from peripheral 2, a peripheral 2 sample is deleted (also shown above). Note: ADC arrival time variations in peripheral 2 are exaggerated above to illustrate both an insertion and a deletion. In practice, at most one correction was made per packet.

Our data alignment approach was to synchronize the timing of each peripheral data sample to the central clock, which, in turn, synchronized them to each other. In addition, even highly accurate clocking on distinct nodes cannot be perfect. That is, the clock rates on distinct nodes are slightly different. Thus, over time, a peripheral ADC produces too many or too few samples, relative to time on the central clock. If clock rates drift, the relative clock rates also drift with respect to each other. Hence, a data alignment algorithm was introduced. This algorithm utilized the timestamp pairs from each peripheral node to maintain an affine synchronization model for each respective peripheral node. The ADC timestamp from each packet per peripheral node was used to estimate the corresponding central node time. This ADC timestamp corresponds to the time of the last sample in the packet; all other sample times within the packet were estimated by subtracting respective multiples of the sampling period (1 ms, in this case). We then compared, on a sample-by-sample basis, when the accumulated temporal drift (i.e., mathematical difference) between the two peripheral clocks was larger than a threshold. For convenience, we assigned the first peripheral as the primary clock. If the second peripheral clock was running more quickly, one data sample was removed from the head of its data stream. If the second peripheral clock was running more slowly, one data sample was interpolated and added to the head of its data stream. In our case, the prior sample value was inserted as the interpolated value. An illustration of this process is shown in Figure 3, and a simple mathematic formulation of this method is available in [49]. A threshold value that was too small (e.g., under a sampling period) led to excessive corrections in which samples were alternately deleted and interpolated in subsequent packets. A threshold value that was too large (e.g., multiple sample periods) allowed larger time synchronization errors to persist longer in the data stream. After some preliminary testing [50] we selected a threshold value of one sample. This approach can be generalized to more than two peripheral nodes.

## 4. Experimental Materials and Methods

**Figure 4:**
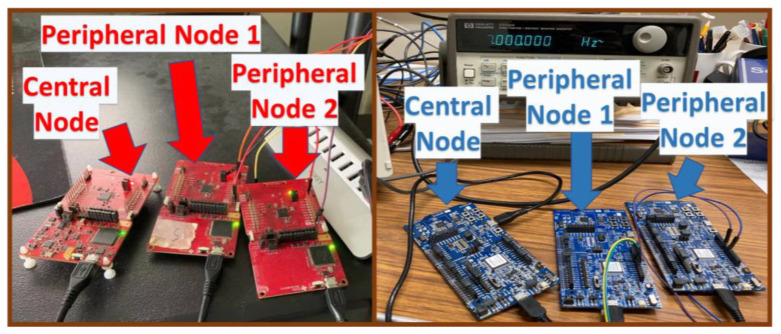
TI platform (**left**) and Nordic platform (**right**).

The TI- and Nordic-based systems, consisting of two peripheral nodes and one central node, were separately implemented and hardware-tested on the benchtop (Figure 4). Both platforms were USB-powered, for convenience. In a fielded system, the peripheral nodes would be battery-powered and physically separated from the central node. The TI CC2642 central node microcontroller was programmed using TI SimpleLink (development kit version 3.10.01.11, compiler version TI v18.12.2 LTS). The TI CC2640R2f peripheral node microcontrollers were programmed using TI SimpleLink (development kit version 1.40.00.45, compiler version TI v16.9.1 LTS). “C” code was developed in TI Code Composer Studio (version 9.0.1). All Nordic microcontrollers were programmed using Nordic nRF52840 SoftDevice (development kit version S140 and SDK version v17.0.0). “C” code was developed in Nordic Segger Embedded Studio (version 5.62). These were the available and supported tools for these two processors available at the time data were collected. For all logged data, offline analysis used MATLAB (the MathWorks, version R2021b).

For each platform, a function generator (HP 33120A) simultaneously applied the same input to one ADC channel of each peripheral node. The generator produced a sine wave ranging from 0.5 V to 2.5 V (1.5 V offset, to align with the unipolar ADCs). During a test trial, the signal frequency was varied from 1 Hz to 12 Hz with an increase of 1 Hz every 1 min. This maximum frequency was selected such that its period (83.3 ms) was well outside our worst-case time synchronization error, since timing errors that are multiples of one sine wave period are ambiguous. Each testing trial was 12 min in duration. Each peripheral node sampled and transmitted these data and the timestamps wirelessly to the central node. For testing, the central node was connected to a PC through a UART port, transferring unsynchronized ADC data packets, their corresponding timestamp pairs, and their ADC final sample timestamps from both peripheral nodes directly into MATLAB in real time. These data were then stored to the hard drive for offline analysis. For each platform, seven trials were collected.

Offline, each 12 min recording from both peripheral nodes for a trial was separately time-aligned, using our time synchronization and data alignment method. Each recording was upsampled by a factor of 100 (via zero insertion followed by lowpass filtering, as implemented by MATLAB “interp”) to improve time resolution between samples from 1 ms to 10 μs. We used zero-phase lowpass filtering in the upsampler; thus, the first and last 10 s of each recording were discarded, to eliminate filter startup/tail transients. For each 12 min trial, 700 s of data remained. The data from each trial were then segmented into 1 s duration contiguous epochs (700 segments/trial × 7 trials = 4900 epochs total). For each epoch, we computed the cross-correlation coefficient function [i.e., normalized such that auto-correlations at zero lag is equal to 1; see the “*normalized*” option of MATLAB’s *xcorr()* function] between the data from the two peripheral nodes, extracting the location of the maximum correlation and the correlation value at this location. All average correlation values exceeded 0.99. The location of the maximum correlation was an estimate of lag/lead between the peripheral ADC channels. The mean and standard deviation lag/lead of the 4900 epochs was reported, and all 4900 values were used for statistical analysis.

The entire process was repeated for all combinations of the number of sequential timestamp pairs used in the affine regression model (N = 2, 4, 8, 16, 32, 64, or 128) and the number of connection intervals between timestamp updates (every 10, 20, 50, and 100 connection intervals). A smaller N is computationally more expedient, but provides less averaging in the least-squares estimate. A small timestamp update period requires more frequent updating of the affine model (thus, computationally expensive), whereas an overly long timestamp update period may not adapt quickly enough to true changes in clock rate.

All statistical comparisons of conditions were computed using SPSS version 28.0.00 (190). The data from each statistical comparison were first tested for normality using the Kolmogorov–Smirnov test. As all data were not normally distributed (*p* < 0.001), a nonparametric Friedman test was used to test performance differences according to the factor timestamp update interval and number of timestamp pairs. If significant, we proceeded to post hoc paired Wilcoxon signed-rank tests with Bonferroni–Holm correction for multiple comparisons. Differences were considered statistically significant for *p* < 0.05.

## 5. Results

**Figure 5:**
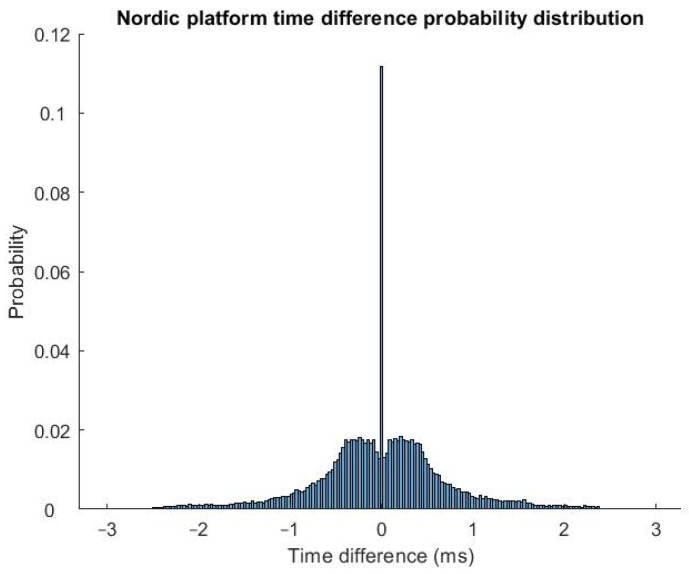
Histogram, scaled as a probability density function estimate, showing Nordic platform time differences between two peripheral nodes. Results are combined from all number of timestamp pairs (N = 2, 4, 8, 16, 32, 64, and 128) and update intervals (100, 200, 500, and 1000 ms).

Figure 5 shows a probability distribution function estimate of inter-channel signed timing errors (positive value corresponding to the first peripheral node leading to the second peripheral node) for the Nordic platform, combining results across all different conditions (update interval = 100, 200, 500, and 1000 ms; N = 2, 4, 8, 16, 32, 64, and 128). Note the large number of times in which the error is equal to 0 lag/lead (count) values (at the upsampled rate). Table 2 (TI) and Table 3 (Nordic) show the average and standard deviation signed and absolute time difference errors (i.e., the absolute value of the signed timing errors) between the two peripheral nodes as a function of different number of sequential timestamp pairs (N) and timestamp update intervals.

### 5.1. Texas Instruments (TI) Platform Results

For the *signed* errors resulting from using the TI platform, the Friedman test (factors: timestamp update interval and number of timestamp pairs) found a statistically significant difference [$\chi^{2}\left(27\right)=622,\;p=6\times 10^{-144}$]. We began post hoc evaluation by identifying the minimum average error within each update interval (i.e., the best as a function of N), identified in bold red font in Table 2. Within the results for each update interval, we pairwise compared (Wilcoxon signed-rank test) the results of this best value of N to each value of N. Significant and insignificant results are shown in Table 2. In most cases, results within an update interval varied with N. Lastly, we compared results from the cell with the overall lowest average error to the best case within each update interval using a Wilcoxon signed-rank test. This lowest average error of 1 ± 228 μs (N = 8 timestamp pairs, 750 ms timestamp update interval) was significantly lower than the others ($p<4\times 10^{-5}$).

We repeated this statistical analysis for the *absolute* errors. The Friedman test found a significant difference [$\chi^{2}\left(27\right)=26,923,\;p=1\times 10^{-311}$]. Post hoc evaluation within each timestamp update interval is shown in Table 2. In all cases, results within an update interval varied with N. The cell with the overall lowest average error was a tie for the 750 ms update interval with N = 2 or 64 (error of 69 ± 71 μs). Between-update interval comparisons with the data corresponding to each overall minimum cell found each to be significantly lower than each of the other minimum cells from the other update intervals ($p<4\times 10^{-13}$).

### 5.2. Nordic Platform Results

For the Nordic platform, the above statistical analysis was repeated. For *signed* errors, the Friedman test found a statistically significant difference between different parameter combinations [$\chi^{2}\left(27\right)=872,\;p=3\times 10^{-166}$]. Post hoc evaluation within each timestamp update interval is shown in Table 3. In most cases, results within an update interval varied with N. The cell with the overall lowest average error of 3 ± 716 μs (N = 64 timestamp pairs, 100 ms timestamp update period) was significantly lower than the others (p<0.0012).

For the *absolute* error, the Friedman test found a significant difference [$\chi^{2}\left(27\right)=108,\;p=1\times 10^{-11}$]. Post hoc evaluation within each timestamp update interval is shown in Table 3. In most cases, results within an update interval did not vary with N. The cell with the overall lowest average error of 477 ± 490 μs (N = 2 timestamp pairs, 200 ms timestamp update period) did not differ significantly from the other minima (p>0.179).

## 6. Discussion and Future Direction

### 6.1. Overall Time Synchronization Performance

When independent wireless peripheral nodes are each collecting ADC data, it is imperative to time-synchronize these data streams. We did so using a BLE implementation from within the application layer, thereby avoiding the need for custom hardware and facilitating software reuse between microcontroller platforms and versions. Our method synchronizes each peripheral to the central clock, thereby mutually synchronizing multiple peripheral nodes. Our method also avoids timestamp “rollover” errors, whereby synchronization remains valid for as long as a device is powered.

We assessed both the signed time synchronization error between two independent peripheral node ADC samples and the absolute error. We tested using input sine wave frequencies spanning 1–12 Hz. The signed error, on average, was quite small, with a best-case mean value of 1 μs for the TI platform (750 ms timestamp update interval, N = 8) and 3 μs for the Nordic platform (100 ms timestamp update interval, N = 64). If this error is large (not the case in these results), then a correctable time bias exists. However, this error can be misleadingly small if approximately half of the errors cause one peripheral to lead, while the other half cause this same peripheral to lag.

Thus, we also assessed the absolute timing error, which better represents performance; it is strongly related to the standard deviation of the signed timing error. Depending on the timestamp update interval (whose possible values varied between platforms) and the number of timestamp pairs (N), the TI platform had mean absolute errors ranging from 69 to 376 μs and a best-case error of 69 ± 71 μs (750 ms timestamp update interval, either N = 2 or N = 64 timestamp pairs). The Nordic platform had mean absolute errors ranging from 477 to 515 μs and a best-case error of 477 ± 490 μs (200 ms timestamp update interval, N = 2 timestamp pairs). These errors had large standard deviations, typically similar in value to the mean. Thus, we also reported the 90th and 95th percentile absolute errors. The 95th percentile errors were less than ∼1 ms for the TI platform and less than 1.8 ms for the Nordic platform, thus being quite comparable. For many engineering applications, these 95th percentile absolute errors likely provide a better design guideline than the other measures.

Surprisingly, these 95th percentile errors did not seem to vary much with timestamp update interval and number of timestamp pairs used in the time synchronization algorithm (see Table 2 and Table 3), although the TI platform may have exhibited somewhat lower 95th percentile errors when using a 750 ms timestamp update interval. Thus, in our work, the added value of averaging (i.e., regressing) over many timestamp pairs may have provided limited value. However, our peripheral and central nodes were located side-by-side in a low-noise laboratory environment. Since timing variations in wireless transmission would seem to represent the largest source of error in our method for generating timestamp pairs, this variation will likely be larger in fielded devices. In that case, averaging timestamp information should prove valuable.

The average ± standard deviation *absolute* errors were larger for the Nordic platform compared to the TI platform. However, we used different connection intervals and, thus, different timestamp update intervals. Hence, we did not compare the platforms statistically. We chose each connection interval as the minimum that performed reliably (i.e., without noticeable packet loss) for the respective platform—15 ms for TI and 10 ms for Nordic. However, it is possible that the longer connection interval for the TI platform led to fewer outlier transmission delays. The few long delays may dominate time synchronization errors (e.g., see Figure 5 and [49]). In opposition to this concern, signal latency is directly proportional to the connection interval. For real-time control applications, shorter latency (and, thus, shorter connection intervals) is desired. Thus, the shorter connection interval for the Nordic platform is advantageous. In addition, the 95th percentile absolute errors were similar between the two systems (each <1.8 ms). Hence, neither platform was clearly better or worse than the other.

Overall, our errors were quite small when considering biomedical signal acquisition. For ECG and EEG (typical sampling rate below 500 Hz), these errors were less than one sample period. For EMG (typical sampling rates of 1000 or 2000 Hz), these errors were 1–4 sampling periods. Hence, our technique improves the design of BLE-based wireless biomedical device systems by facilitating data alignment that is precise enough for many applications, is low-latency and high-throughput, and is transferable between manufacturer devices.

Previous time synchronization methods developed specifically for BLE systems reported somewhat lower average ± standard deviation time synchronization errors; however, as detailed above, these systems are not transferrable or suitable for continuous, high-throughput and low-latency applications as supported by our method. When custom hardware is added to BLE systems, errors as low as 9 ± 17 µs have been achieved [40]. Such systems are not readily transferrable to different microcontrollers or microcontroller versions. When systems are time-synchronized when a connection is established, timing errors as low as 40 ± 14 µs have been demonstrated [38]. However, this method, as well as those which utilize the BLE beacon role [37], drift over time and/or are not compatible with continuous high-throughput data acquisition. Our method fills a role not currently provided by these other techniques.

### 6.2. Robustness of the Timestamps

Synchronization is based entirely on the precision and robustness of the timestamps. When referring to precision, our method relies more on the repeatability of the timestamps, rather than their accuracy. For example, the peripheral $TS_{ADC}$ timestamp is created and associated with the final ADC sample in a packet. However, this clock query is completed after the final ADC sample has been acquired and within the resulting ADC software interrupt service routine. In other words, this timestamp always represents a time that is slightly delayed from the actual time at which that last ADC sample is converted. However, this time difference should be small (a few μs). More importantly, this time difference should be very similar on the two peripheral nodes. So long as both peripheral nodes experience the same repeatable delay, their synchronization is preserved.

More concerning is the central timestamp $TS_{C}$, which is generated by querying the central clock after peripheral data are received and then adding one connection interval to this value. As noted above, the precision of this timestamp depends on the reliability of wireless transmission from the peripheral to the central and then (at the next connection interval) from the central node to the peripheral node. These external delays should be less reliable; hence, our use of a synchronization algorithm to average out the timestamp data from several update intervals via regression.

Our laboratory environment happened to have few other active BLE devices, resulting in the Bluetooth 2.4 GHz transmission frequency band experiencing limited use. Thus, we experience limited “blocked” transmissions. A blocked transmission occurs when a given wireless frequency channel is in use; hence, the scheduled transmission does not have channel access. When a BLE transmission is blocked, BLE waits an additional connection interval and then reattempts transmission. This action is not reported to the application layer software. When a central to peripheral transmission is blocked and delayed by one connection interval, the central clock timestamp becomes stale (incorrect) by one connection interval in our scheme. The connection interval (10 or 15 ms, depending on the platform) is much longer than our average absolute errors. We anecdotally found much higher absolute errors during the few times in which transmission was blocked. In other more complex laboratory or field settings, this issue may be much more prevalent [51].

### 6.3. Limitations and Future Work

Because blocked central to peripheral transmissions lead to large but predictable errors in the central timestamp $TS_{C}$, they likely can be detected and corrected. In particular, the errors are approximately a multiple of the connection interval. Finding such errors on our available dataset was rather limited, since our rate of blocked transmissions was quite small (estimated below 0.001%). Thus, we simulated this condition offline in MATLAB. We created 1 h of central timestamps at equal timestamp intervals of 100 ms. We then created the matching peripheral timestamps with a time offset error drawn from an independent, random, uniform distribution ranging from 0 to 1.25 ms. This span is representative of the errors found in our TI and Nordic platforms. Lastly, we treated each central timestamp as an independent Bernoulli trial, adding a 10 ms delay (representing a blocked transmission) with a selection probability of 0.1%. Hence, on average, one in every thousand central timestamp updates was treated as having been blocked. This blocking rate is artificially high compared to our dataset, but useful in simulation. We then independently analyzed our timestamp pairs to detect blocked transmissions. To do so, we formed the ratio, $Ratio\left[m\right]$, of the difference of the last two central timestamps to the difference of the last two peripheral timestamps, as follows:

$$Ratio\left[m\right]=\frac{TS_{C}\left[m\right]-TS_{C}\left[m-1\right]}{TS_{P}\left[m\right]-TS_{P}\left[m-1\right]}.$$

Whenever this ratio was greater than 1.5, we correctly detected every blocked transmission, with no false positives. Of course, detection is likely more complex in practice. In particular, a transmission can be blocked for several transmit cycles.

Another limitation is that we only evaluated synchronization performance in systems using two peripheral nodes. As more peripheral nodes are included, the connection interval for each peripheral node will likely need to grow. Doing so will increase packet size (more ADC samples per packet due to the longer interval), which may eventually exceed channel capacity. Longer connection intervals also increase latency, which is detrimental for various applications, including real-time control (e.g., prostheses and orthoses) and biofeedback. Methods to mitigate these limitations include wiring multiple sensors to one node (thus decreasing the number of required nodes) and compression of ADC data samples. Each of these possibilities can be studied as future work.

While we evaluated performance as a function of the number of timestamp pairs and timestamp update interval, many other parameters/configurations could be evaluated. To mitigate this limitation, we suggest future studies. Other parameters include packet size, ADC sampling rate, and connection interval (in cases where the minimum interval is not necessary or desired). Configuration considerations include the distance between nodes and number of other devices competing for BLE spectrum. Furthermore, operational schemes that operate even in the presence of inevitable packet loss should be evaluated.

## 7. Conclusions

We developed a time synchronization algorithm and data alignment method that operates at the BLE application layer, for low-latency, high-throughput applications. Compared to other methods, this method is easily transferred from one BLE platform to another, as demonstrated herein on two platforms. The method was implemented without the costs associated with specialized hardware. Our best performance achieved absolute time differences between two independent peripheral nodes of 69 ± 71 μs for the TI platform and 477 ± 490 μs for the Nordic platform on average. The 95th percentile absolute errors for both TI and Nordic platforms were less than 1.8 ms, which is appropriate for use by most ECG, EEG, and EMG applications. The 95th percentile results were, contrary to our original hypothesis, not particularly sensitive to the timestamp update interval or the number of timestamp pairs used in the time synchronization model. Additional evaluation is warranted in environments in which delayed or blocked Bluetooth transmissions are likely, i.e., situations not considered by our research but relevant to practical usage. Although the method should scale to systems with many peripheral nodes, evaluation in such systems is an appropriate next step.

## Figures and Tables

**Table 1:** Summary of existing time synchronization methods for BLE and their limitations for use with low-latency, high-throughput applications that are transferrable between different manufacturer microcontrollers. The MAC layer refers to the medium access control layer of the communications protocol.

Method Name	Protocol Used	Reported Time Precision	Limitation for Our Application	References
Bluesync	BLE Beacon	451 ± 4.5 μs	Not continuous synchronization	[37]
Bideaux et al.	BLE Beacon	7.5 ± 0.4 ms; 40 ± 14 μs	Not continuous synchronization	[38]
CheapSync	BLE Beacon	∼10 μs	Not continuous synchronization	[39]
Rheinlander and Wehn	BLE Beacon	<20 μs	Requires additional hardware	[40]
Dian et al.	BLE Advertise	∼700 μs	Not continuous synchronization	[41]
NTP	NTP	∼10 μs	Large message volume and computation	[43]
TTS	TTS	∼10 μs	Requires fast two-way messaging	[44]
TPSN	TPSN	∼10 μs	Programmed at MAC layer	[45,46]
FTSP	FTSP	∼10 μs	Programmed at MAC layer	[31]

**Table 2:** Summary results for TI microcontroller system. Cells in bold red font indicate results with the minimum mean value within that timestamp update interval. “NS” (in bold blue font) denotes that the results in this cell are NOT significantly different from those of the cell with the minimum mean value within that timestamp interval. Mean ± SD results are each from 4900 epochs. The last two columns list 90th and 95th percentile absolute errors.

Timestamp Update Interval (ms)	Number of Timestamp Pairs (N)	Mean ± SD Signed Errors (μs)	|------- Absolute Errors -------|		
			Mean ± SD (μs)	90th % (ms)	95^th^ % (ms)
150	2	13 ± 508	348 ± 370	0.72	0.91
	4	37 ± 442	**318 ± 309**	0.70	0.83
	8	26 ± 467	349 ± 314	0.75	0.92
	16	17 ± 502	365 ± 344	0.74	0.91
	32	14 ± 495	366 ± 333	0.85	1.05
	64	33 ± 490	376 ± 316	0.85	1.03
	128	**11 ± 439**	336 ± 283	0.68	0.87
300	2	**7 ± 483** **NS**	359 ± 323	0.80	0.94
	4	27 ± 517	373 ± 359	0.80	0.91
	8	12 ± 476	346 ± 327	0.75	0.96
	16	27 ± 458	357 ± 288	0.75	0.91
	32	11 ± 434	322 ± 292	0.74	0.90
	64	9 ± 412 **NS**	317 ± 263	0.63	0.85
	128	**4 ± 412**	**305 ± 277**	0.75	0.90
750	2	18 ± 97	**69 ± 71**	0.18	0.19
	4	18 ± 219	114 ± 188	0.22	0.53
	8	**1 ± 228**	115 ± 197	0.22	0.44
	16	12 ± 267	153 ± 218	0.42	0.59
	32	23 ± 200	120 ± 162	0.22	0.41
	64	18 ± 97	**69 ± 71**	0.18	0.19
	128	8 ± 197	106 ± 166	0.22	0.45
1500	2	57 ± 413	301 ± 288	0.70	0.85
	4	18 ± 335 **NS**	241 ± 233	0.57	0.70
	8	**7 ± 314**	201 ± 241	0.54	0.74
	16	65 ± 361	249 ± 269	0.65	0.76
	32	22 ± 317	196 ± 250	0.53	0.75
	64	27 ± 355	217 ± 282	0.64	0.78
	128	13 ± 282 **NS**	**167 ± 227**	0.46	0.66

**Table 3:** Summary results for Nordic microcontroller system. Cells in bold red font indicate results with the minimum mean value within that timestamp update interval. “NS” (in bold blue font) denotes that the results in this cell are NOT significantly different from those of the cell with the minimum mean value within that timestamp interval. Mean ± SD results are each from 4900 epochs. The last two columns list 90th and 95th percentile absolute errors.

Timestamp Update Interval (ms)	Number of Timestamp Pairs (N)	Mean ± SD Signed Errors (μs)	|------- Absolute Errors -------|		
			Mean ± SD (μs)	90th % (ms)	95th% (ms)
100	2	40 ± 731	513 ± 522	1.26	1.66
	4	54 ± 700	491 ± 501 **NS**	1.17	1.57
	8	41 ± 700	**488 ± 504**	1.17	1.54
	16	53 ± 714	495 ± 517 **NS**	1.20	1.63
	32	13 ± 709	494 ± 509 **NS**	1.20	1.63
	64	**3 ± 716**	502 ± 510 **NS**	1.26	1.63
	128	29 ± 701	491 ± 501 **NS**	1.17	1.60
200	2	48 ± 682	**477 ± 490 **	1.16	1.57
	4	64 ± 705	491 ± 509 **NS**	1.17	1.57
	8	33 ± 709	488 ± 515 **NS**	1.23	1.63
	16	**4 ± 714**	498 ± 511 **NS**	1.23	1.63
	32	36 ± 707	492 ± 509 **NS**	1.23	1.60
	64	58 ± 99	493 ± 504 **NS**	1.20	1.54
	128	27 ± 708 **NS**	489 ± 513 **NS**	1.17	1.60
500	2	48 ± 700	492 ± 499 **NS**	1.17	1.60
	4	**27 ± 710**	495 ± 509 **NS**	1.20	1.55
	8	55 ± 731	515 ± 522	1.26	1.72
	16	41 ± 715	491 ± 521 **NS**	1.20	1.63
	32	45 ± 712 **NS**	504 ± 505 **NS**	1.20	1.60
	64	82 ± 712	499 ± 514 **NS**	1.20	1.61
	128	71 ± 696	**486 ± 503**	1.14	1.57
1000	2	49 ± 699	492 ± 499 **NS**	1.17	1.60
	4	**28 ± 710**	496 ± 509 **NS**	1.20	1.54
	8	56 ± 731	515 ± 522	1.26	1.72
	16	42 ± 716	492 ± 522 **NS**	1.20	1.63
	32	44 ± 712 **NS**	504 ± 505 **NS**	1.20	1.60
	64	80 ± 712	499 ± 515 **NS**	1.20	1.63
	128	71 ± 696	**487 ± 502**	1.14	1.57

## Data Availability

The data presented in this study are available on request from the corresponding author.
